# Mechanical signatures of human colon cancers

**DOI:** 10.1038/s41598-022-16669-3

**Published:** 2022-07-21

**Authors:** Evelyne Lopez-Crapez, Luca Costa, Guillaume Tosato, Jeanne Ramos, Thibault Mazard, Janique Guiramand, Alain Thierry, Jacques Colinge, Pierre-Emmanuel Milhiet, Christine Bénistant

**Affiliations:** 1 Institut Régional du Cancer Montpellier (ICM), Montpellier, France; 2 Institut de Recherche en Cancérologie de Montpellier (IRCM), INSERM U1194, Université de Montpellier, Montpellier, France; 3 Unité de Recherche Translationnelle, Institut du Cancer de Montpellier (ICM), Montpellier, France; 4 Centre de Biologie Structurale (CBS), CNRS, INSERM, Université de Montpellier, Montpellier, France; 5 Centre Hospitalier Universitaire (CHU) Lapeyronie, Montpellier, France; 6 Institut des Biomolécules Max Mousseron (IBMM), UMR5247 CNRS, Université de Montpellier, ENSCM, Montpellier, France

**Keywords:** Biophysics, Cancer

## Abstract

Besides the standard parameters used for colorectal cancer (CRC) management, new features are needed in clinical practice to improve progression-free and overall survival. In some cancers, the microenvironment mechanical properties can contribute to cancer progression and metastasis formation, or constitute a physical barrier for drug penetration or immune cell infiltration. These mechanical properties remain poorly known for colon tissues. Using a multidisciplinary approach including clinical data, physics and geostatistics, we characterized the stiffness of healthy and malignant colon specimens. For this purpose, we analyzed a prospective cohort of 18 patients with untreated colon adenocarcinoma using atomic force microscopy to generate micrometer-scale mechanical maps. We characterized the stiffness of normal epithelium samples taken far away or close to the tumor area and selected tumor tissue areas. These data showed that normal epithelium was softer than tumors. In tumors, stroma areas were stiffer than malignant epithelial cell areas. Among the clinical parameters, tumor left location, higher stage, and RAS mutations were associated with increased tissue stiffness. Thus, in patients with CRC, measuring tumor tissue rigidity may have a translational value and an impact on patient care.

## Introduction

Colorectal cancer (CRC) is the third cancer in terms of incidence and the second cause of cancer-related death worldwide^1^. This is partly explained by the fact that CRC is often detected at late stages when the disease has spread to distant organs. Thus, it is important to identify new biomarkers for the early diagnosis of CRC and to develop more effective treatments.

In the clinic, CRC are usually classified in: (1) proximal or right-sided when they originate from colon sections proximal to the splenic flexure (cecum, ascending colon and transverse colon); (2) distal or left-sided when they are in the descending colon or sigmoid colon; and (3) rectal cancers when they are within 15 cm of the anal sphincter. Many data now stress the importance of the tumor location for CRC prognosis and management^2–4^.

Currently, CRC prognostic evaluation is based on the tumor node metastasis (TNM) staging system by the American Joint Committee on Cancer. The 5-year survival rate is ~ 93% for patients with stage I CRC, ~ 80% and 60% for patients with stage II and stage III CRC, respectively, and ~ 14% for patients with metastatic CRC. CRC management is mainly based on the cancer stage: surgery alone for localized CRC, and surgery followed by adjuvant chemotherapy for stage III CRC. However, the selection of patients with stage II CRC that may benefit from chemotherapy is still debated and biomarkers are needed to identify patients with stage III tumors at low risk of recurrence^5^.

Genetic alterations of CRC cells have been associated with tumor progression^6^ and are used for treatment decision-making^7^. In the canonical pathway of carcinogenesis, the starting point is generally a mutation in the *APC* tumor suppressor gene, and progression is dependent on the sequential accumulation of mutations mainly in the *KRAS*, *NRAS*, *BRAF*, *TP53* and *PI3KCA* genes. Moreover, *KRAS* and *NRAS* mutational status can predict the response to anti-EGFR therapies.

Besides these clinical and molecular characteristics, no other factor related to the local microenvironment is taken into account. However, the physical properties of the cells and of their microenvironment could influence cancer progression and metastasis formation^8^. For example, it was reported that extracellular matrix (ECM) stiffening strongly supports normal mammary cell reprogramming into malignant cells by the RTK/RAS oncogenes^9^ and plays an important role in breast cancer cell migration through matrices^10^. The shape and linearity of collagen fibers in the tumor microenvironment also is a prognostic factor of survival^11^. Finally, it has been shown that the microenvironment compaction prevents drug access and immune cell infiltration^12^. Although biological tissues appear as macroscopically solid objects they do not behave as prefect elastic. Their mechanical behaviors include a complex, time and rate-dependent combinaison of viscoelasticity, proelasticity, plasticity and nonlinear elasticity that can be measured using several technics^10,13^. One frequent parameter used to characterize tissue mechanical properties is the elastic or Young modulus originally defined for homogeneous materials. It is however used as an average Young's Modulus to quantify the elastic deformation of single cells^14^ and tissues^15,16^. Young's moduli of human tissues vary from 100 s of Pascal (Pa) for brain and fat tissues to 10 s of GPa in bone^10^. To our knowledge, little is known about human colon Young’s moduli and to its associated changes during cancer progression. Nevertheless, we know that CRC cells are sensitive to mechanically-induced forces, such as the compression induced by the primary tumor on the surrounding cells that favors their transformation^17^. They also remodel their ECM with the progressive linearization and degree of organization of fibrils from healthy to perilesional and CRC ECM associated with a steady increase in stiffness and collagen cross-linking. In the perilesional ECM, these changes occurred concomitantly with increased vascularization, whereas in neoplastic ECM, they were associated with altered modulation of matrisome proteins and increased content of hydroxylated lysine and lysyl oxidase^18^.

Atomic force microscopy (AFM) is a type of scanning probe microscopy that is generally used to study the sample topography at the nanoscale. In AFM, a sharp tip connected to a small flexible spring-like cantilever is displaced over the sample thanks to piezo-electric elements. The measurement of the cantilever deflection allows the accurate evaluation of the interaction forces between the tip and the sample. Additionally, it is possible to probe the sample mechanical properties through the mechanical contact with the tip. By recording the deflection of a calibrated probe, AFM is an outstanding tool to measure the sample mechanical responses over a range compatible with biological specimens in physiological conditions. For example, flexible cantilevers with a tip (i.e. a silica bead with a µm diameter) can be used to probe the mechanical properties of cells and tissues. Upon characterization of the cantilever stiffness and the bead diameter, it is possible to calculate the local Young’s modulus with appropriate mathematical models (for example the Hertz model for a spherical bead)^19^. This approach was previously used to assess the stiffness of human breast cancer tissues^15^. It was also used to study the rigidity of human colon in a restricted number (3–4) patients using either dried and rehydrated, fresh or frozen tissues^13,18,20,21^. Recently, a complete protocol to analyze CRC tissues has been published^21^ and used to determine the role of the mechanical microenvironment during angiogenesis of CRC metastases in liver^22^. Here, we improved this latter protocol using a camera to precisely place the cantilever tip over the region of interest (ROI) in order to characterize the Young Moduli of healthy colon and CRC tissue samples in a larger cohort and to correlate these values with clinical and molecular parameters.

## Materials and methods

### Patients and tissue samples

**Figure 1:**
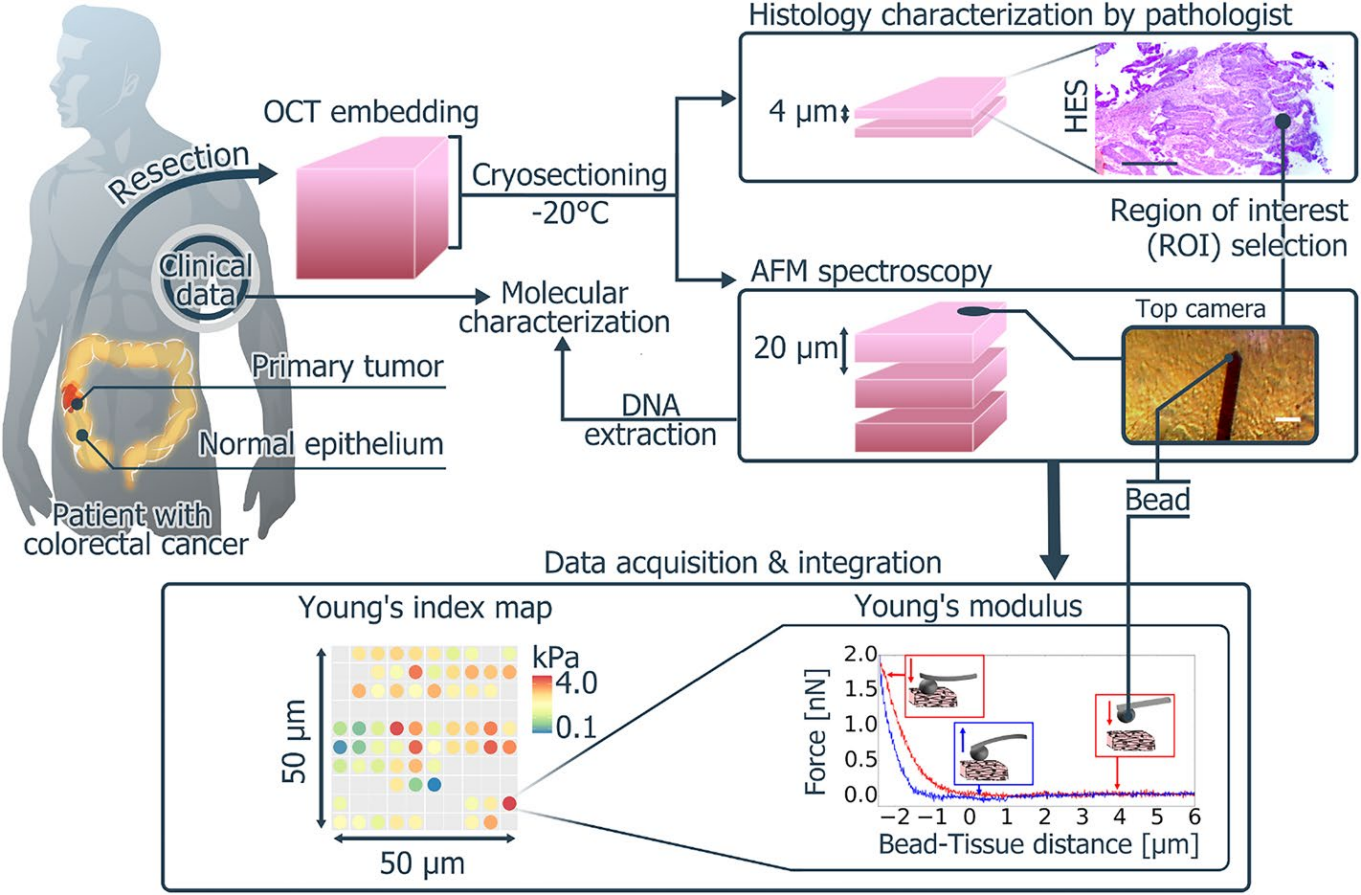
Flow chart of the experiments. First, normal and tumor tissues are harvested during surgery and good-quality tissue specimens are selected by the pathologist. After OCT embedding, samples are frozen in liquid nitrogen and stored at − 80 °C. Frozen samples are then cut: two 4 µm cryosections are used for histology (one for HES and one for Picro-Sirius red staining), and three 20 µm sections for AFM analysis. The AFM JPK Nanowizard 4 microscope is equipped with a top view camera to visualize the tissue slides. The AFM head is placed over the tissue slide that is immersed in PBS containing anti-proteases. The cantilever with a 10.2 µm silica sphere is calibrated in a liquid solution, and placed above the ROI selected on the tissue slide (bar = 25 µm). Force curves are acquired on a 50 µm × 50 µm grid (red curve: probe approach; blue curve: probe retraction) using a 2 nN indentation force at 2 µm/s speed to induce 2–3 µm indentations. The elastic Young’s modulus is calculated by fitting the probe approach curve of the indentation cycle with the Hertz contact model. Insets show the tip approaching and retracting from the tissue. Finally, Young’s modulus measurements in relation to the coordinates in the map are generated and used for the analysis. The figure was created using the free and open source software Inkscape 1.0.2.

Tissue samples were from a prospective cohort of 18 patients with colon cancer accrued within the Clinical and Biological Database BCB COLON (Institut du Cancer de Montpellier-Val d’Aurelle, France, Clinical trial Identifier ICM-BB-0033–00059 #NCT03976960) from October 2018 to December 2019. The study protocol was approved by the French Ethics Committee CPP (Comité de Protection des Personnes) Sud Méditerrannée III (Ref#2014.02.04) and by the Montpellier Cancer Institute Review board (ICM-CORT-2017-14). The study complied with all relevant ethical regulations for working with human participants, and a written consent was obtained for all patients. All patients were chemotherapy-naïve at sample collection. For each patient, paired samples of the primary tumor and of macroscopically colon normal tissue, excised at a distance from the tumor, were selected by the pathologist during extemporaneous examination. Each sample was immediately embedded in Tissue-Tek^®^ OCT (Sakura Finetek France SAS) using disposable plastic Cryomold^®^ molds and stored at − 80 °C until analysis. Cryosections were cut at − 20 °C using a microtome (Microm, HM 550). Tissue sections were immediately absorbed on coated microscope slides (FLEX IHC, DAKO) and stored at − 80 °C. Two thin sections of 4 µm were used for histology characterization and quality control analysis after hematoxylin, eosin and saffron (HES) staining or Picro-Sirius red staining, and three consecutive thick sections of 20 µm were used for AFM studies (AFM slides) (Fig. 1).

The patients’ demographic and clinical characteristics, such as sex, age, tumor location and molecular characterization (microsatellite instability/MSI, RAS and BRAF status) were extracted from their medical records. When molecular information was not available, particularly for patients without metastatic disease (mutational status) and for > 60-year-old patient (MSI status), DNA was directly extracted from the AFM slides using the DNA QIAamp DNA Extraction Kit (Qiagen), according to manufacturer’s instructions, after the stiffness measurement by AFM. *KRAS* exon 2–4, *NRAS* exon 2–4 and *BRAF* exon 15 sequences were analyzed after PCR amplification according to the previously described methodology^23^. MSI status was determined as previously described^24^. After confirming the tissue quality (well-preserved histological structures) after cryopreservation, the AFM analysis was focused on section areas selected by the pathologist by microscopy observation of the HES-stained slides. Regions of interest (ROI) that contained normal tissue, mainly malignant epithelial cells, or mainly microenvironment (stroma) cells were highlighted with circles. Collagen content in ECM was evaluated by the pathologist through examination of Picro-Sirius red-stained sections under a polarized light microscope.

### AFM spectroscopy

AFM is an outstanding tool to locally probe sample stiffness^25^; however, very few studies are available on tissue rigidity measurement. We think that two conditions are required to obtain reliable data: (1) tissue integrity should be preserved as much as possible and (2) tissue sections must be thick enough to avoid the mechanical contribution of the rigid glass substrate during Young’s modulus measurement.

Ideally, fresh tissue samples within 8 h of surgery should be used, but this is not possible logistically in clinical settings. Therefore, tissue samples that were directly snap frozen in liquid nitrogen after embedding in OCT were used. This methodbetter preserved the tissue ultrastructure, facilitated slicing compared with directly nitrogen-frozen samples, and increased the tissue section adhesion to the slides (data not shown). Similar results were recently reported in a study that compared fresh and OCT-embedded frozen CRC samples^19^ (Fig. 1).

Concerning the substrate contribution in AFM measurements^26^, although it can be taken into account using mathematical correction models^27^, 20 µm thick tissue sections were used as described previously^21^ (Fig. 1). Indeed, in experimental conditions where the maximal indentation during a cycle represents 10% of the tissue thickness (i.e. 2–3 µm), the substrate contribution can be considered negligible and the Young’s modulus of the tissue can be correctly evaluated.

Just before AFM data acquisition, slides were rapidly thawed by three washes in room-temperature PBS containing anti-proteases (cOmplete™, EDTA-free Protease Inhibitor Cocktail, Roche, France), and kept in this solution for all measurements to better preserve tissue integrity. ROIs selected on HES-stained slides were reported on the back of the AFM slides. Using the top view camera, the cantilever tip was centered on the ROI.

AFM spectroscopy analysis^28^ was performed using a JPK Nanowizard 4 microscope equipped with a CellHesion stage (JPK, Berlin, Germany) to increase the length of each indentation cycle up to 50 µm (i.e. the distance needed to detach the cantilever tip from steaky tissues). For this study, CP-CONT-SIO-D cantilevers with 10.2 µm colloidal beads as tips were used (NanoAndMore, France). Indeed, as normal human colon epithelial cells have a columnar shape with an average height of 30 µm and a width of about 10–15 µm, only one or two cells could be in contact with a bead of this size during measurements. Therefore, this bead size should allow eliminating differences in intracellular stiffness^29^, and minimizing the indentation cycle heterogeneity. Spherical beads were chosen as AFM tips because indentation cycles performed with spherical indenters are well modeled by the Hertz model^15^ that can be used to evaluate the Young’s modulus. Several AFM tips (beads) were used for each sample to minimize the influence of tip contamination on the Young’s modulus measurements. Cantilever stiffness and optical lever sensitivities were calibrated in a liquid environment using the Contact-Free-Method provided by JPK AFM, and based on a mix of the thermal and Sader^30^ calibration methods. The calibrated spring constant for cantilevers was 0.15–0.24 N/m. AFM spectroscopy indentation cycles were performed using a 2 nN as maximum force threshold (i.e. the force to be applied to obtain an indentation depth that corresponded to 10% of the tissue thickness) at a loading rate of 2 µm/s. This allows acquiring hundred curves in ~ 40 min. A squared grid of 10 × 10 pixels covering a region of 50 × 50 µm was fixed to the ROI to define a force map constituted by 100 indentation curves. For each AFM slide (1–3 AFM slides per patient), 2–7 force maps were acquired in the selected ROI using 1–3 probes. When three slides were used, 7500 µm^2^ of tissue could be probed.

The AFM was equipped with a top view camera with a 12X zoom lens and a coaxial LED illumination system (Navitar) to visualize the AFM cantilever and the tissue sections for precise position of the cantilever tip (Fig. 1).The acquired data were analyzed with the JPK AFM data processing software. Force curves were excluded when (1) the tip/cantilever was in permanent contact with the tissue during the whole indentation cycle; (2) the contact point was not well defined (e.g. curves that are not flat over at least 2 µm before the mechanical contact point between tip and tissues); and (3) the curves were too noisy: oscillating with a noise in the force channel comparable with the indentation maximal threshold force. After quality filtering all measurement points, reliable 50 × 50 µm maps were obtained (Fig. 1). For the selected curves, the elastic Young’s modulus (E; Pa) was evaluated by fitting each force versus the tip-tissue distance curve with the Hertz contact model for indenting an infinite isotropic elastic half space with a solid sphere, as previously described^31^.

### Statistical analyses

Descriptive data are presented with median and interquartile range (IQR) for quantitative variables and with frequency and proportions for qualitative variables (Table 1). Spatial and statistical analyses were done using R 4.0.3.

**Table 1:** Patients’ characteristics (MSS, microstaellite stable; MSI, microsatellite instability; WT, wild type).

	Number	Percentage
**Age (years)**		
Mean ± SD	67.6 ± 9.7	
Median	65	
Range	55–85	
**Sex**		
Male	7	38.9
Female	11	61.1
**CRC location**		
Right colon	10	55.6
Left colon	8	44.4
**Mucinous component**		
Yes	8	44.4
No	10	55.6
**Lymphovascular infiltration**		
Yes	10	55.6
No	8	44.4
**Perinervous infiltration**		
Yes	5	27.8
No	13	72.2
**Stage**		
I	1	5.5
II	10	55.6
III	3	16.7
IV	4	22.2
**MSI status**		
MSS	9	50
MSI	9	50
**Mutation status**		
WT	6	33.3
Mutated (1 mutation)	11	61.1
Mutated (at least 2 mutations)	1	5.8
**Mutation type**		
KRAS mutated	7	58.3
NRAS mutated	1	8.4
BRAF mutated	4	33.3

- Spatial pattern and map coverage
  Each map was represented using the *geog4g3* R package based on coordinates (example in Supplementary Fig. 1A) and analyzed using the *spatstat* R package. As data followed a gamma distribution with several values close to 0.1 kPa, a square root transformation was used to obtain a readable color-scale. Point patterns were used for density and distance matrix generation (Supplementary Fig. 1A,B). Then, the mean of the minimal distances between a given point and all the others in the distance matrix were computed. This mean minimal value was used to characterize map coverage (Supplementary Fig. 1C,D). Then, the map coverage was represented using a distance index between points (see S Supplementary Fig. 1): higher index values indicated lower map coverage. This index was not significantly different among tissue types (weighted ANOVA $F=1.373; p=0.249$). Therefore, all samples were pooled (Supplementary Fig. 1D) and by visual inspection, a distance index threshold of 8 was chosen to retain only the maps with adequate coverage. The spatial pattern of the selected maps representative of the different tissues and tissue zones analysed are presented in Supplementary Fig. 1E.
- Generalized mixed effects model
  The 50 µm × 50 µm maps show repeated measurements of the elastic Young’s modulus at various locations. As the presence of biological and technical noise in these data might induce type I autocorrelation, a generalized linear mixed model (GLMM)^32^ with a penalized quasi-likelihood (PQL) was used because data were not normally or log-normally distributed^33^. The *glmmPQL* function from the *MASS* R package was used with Gamma distribution and log link. Furthermore, as several maps were available for each patient, the intra-patient, inter-patient and inter-tissue variability could be studied. For the intra-patient variability, patients were considered as fixed effect and the maps of a patient as random effect (Table 2). For inter-patient variability, patients and maps were considered as random effects (Table 2). For inter-tissue variability, patients, maps, and cross-effects between maps and tissue types were considered as random effects, and the tissue type as fixed effect (Table 3). This approach for dealing with a small patient cohort was previously validated^34^. GLMMPQL is a widely-used model to study spatial distribution in ecology, geology, and environmental exposure^35^ with data displaying the same statistical properties as our data. Specifically, the residual errors after application of random effects follow a normal distribution in an homoscedastic manner.

**Table 2:** Intra-individual and inter-individual variability in colon tissue stiffness.

Spatial model (GLMMPQL)		
Covariates	$F$-value	$P$ (Wald)
**Distal epithelium**		
Map	0.175	0.676
Patient	163.3	0.006
**Proximal epithelium**		
Map	22.4	0.014
Patient	3.1	0.079
**Tumor epithelium**		
Map	5.2	0.054
Patient	21.4	0.002
**Stroma**		
Map	140.5	0.001
Patient	29.6	0.009
**Mixed**		
Map	48.6	< 0.0001
Patient	90.4	< 0.0001

Results of generalized linear mixed models fitted with multivariate normal random effects (GLMMPQL) for intra-individual variability (Map: fixed effect) and inter-individual variability (Patient: fixed effect) analysis with the Wald *F* test and the obtained $P$-values.

**Table 3:** Summary of the paired comparisons of all colon tissue subtypes using glmmPQL.

	Spatial model (GLMMPQL)			
	$\beta$ coefficient estimation	SD	$t$*-value*	$P$
**Reference: Distal epithelium**				
Proximal epithelium	0.797	0.944	0.844	0.446
**Reference: Normal tissue (PE + DE)**				
Tumor Epithelium	0.192	0.078	2.44	0.0146
Stroma	2.18	0.445	4.91	< 0.0001
Mixed	1.75	0.415	4.214	< 0.0001
**Reference: Tumor epithelium**				
Stroma	1.249	0.329	3.8	0.0002
Mixed	0.969	0.216	4.486	< 0.0001
**Reference: Mixed**				
Stroma	0.407	0.283	1.437	0.1578
T + S	− 0.164	0.237	-0.692	0.4921

Results (tissue type: fixed effect) are presented with the estimated $\beta$ parameter, standard deviation (SD), and the $t$ test value with the associated $P$-value. Intra-individual variability (Map: fixed effect) and inter-individual variability (Patient: fixed effect) were taken into account and are summarized in Supplementary Table S1.

*PE* proximal normal epithelium, *DE* distal normal epithelium, *T* tumor epithelium, *S* stroma, *Mixed* tumor areas that contain both tumor epithelial and stromal cells.
  To evaluate fixed effects, the Wald *F* test was performed for each parameter of the models due to data overdispersion^33^. Then, the model statistical significance was assessed using the ANOVA likelihood ratio and Wald *F* tests.

### Ethical approval

Samples were collected in the framework of the Clinical and Biological Database BCBCOLON (Institut du Cancer de Montpellier-Val d’Aurelle, France, Clinical trial Identifier #NCT03976960). The protocol was approved by the French Ethics Committee CPP (Comité de Protection des Personnes) Sud Méditerrannée III (Ref#2014.02.04) and by the Montpellier Cancer Institute Review board (ICM-CORT-2017-14).

### Informed consent

We complied with all relevant ethical regulations for work with human participants, and informed written consent was obtained from all patients.

## Results

### Patient cohort

We characterized the stiffness of healthy and malignant colon tissues from a cohort of 18 patients with CRC naïve of treatment (Table 1) by AFM-based mapping of 96 areas. The cohort included 11 women and 7 men (8 CRC in the left colon and 10 in the right colon). Their mean age was 67 years, within the reported frequency peak of CRC (60–70 years of age)^36^. Most CRC were stage II (n = 10). Moreover, 44.5% of CRC harbored RAS mutations, in agreement with the literature, and 87.5% of RAS mutated tumors displayed a *KRAS* exon 2 single base substitution. MSI tumors (n = 9; 50%) were overrepresented in our cohort because MSI is detected in ~ 15% of all CRC^37^. After removing the maps with a distance index < 8, the median number of maps per patient was 4 (range 1–8) and the median number of values per map was 56 (range 21–93).

### Stiffness of normal colon epithelium

**Figure 2:**
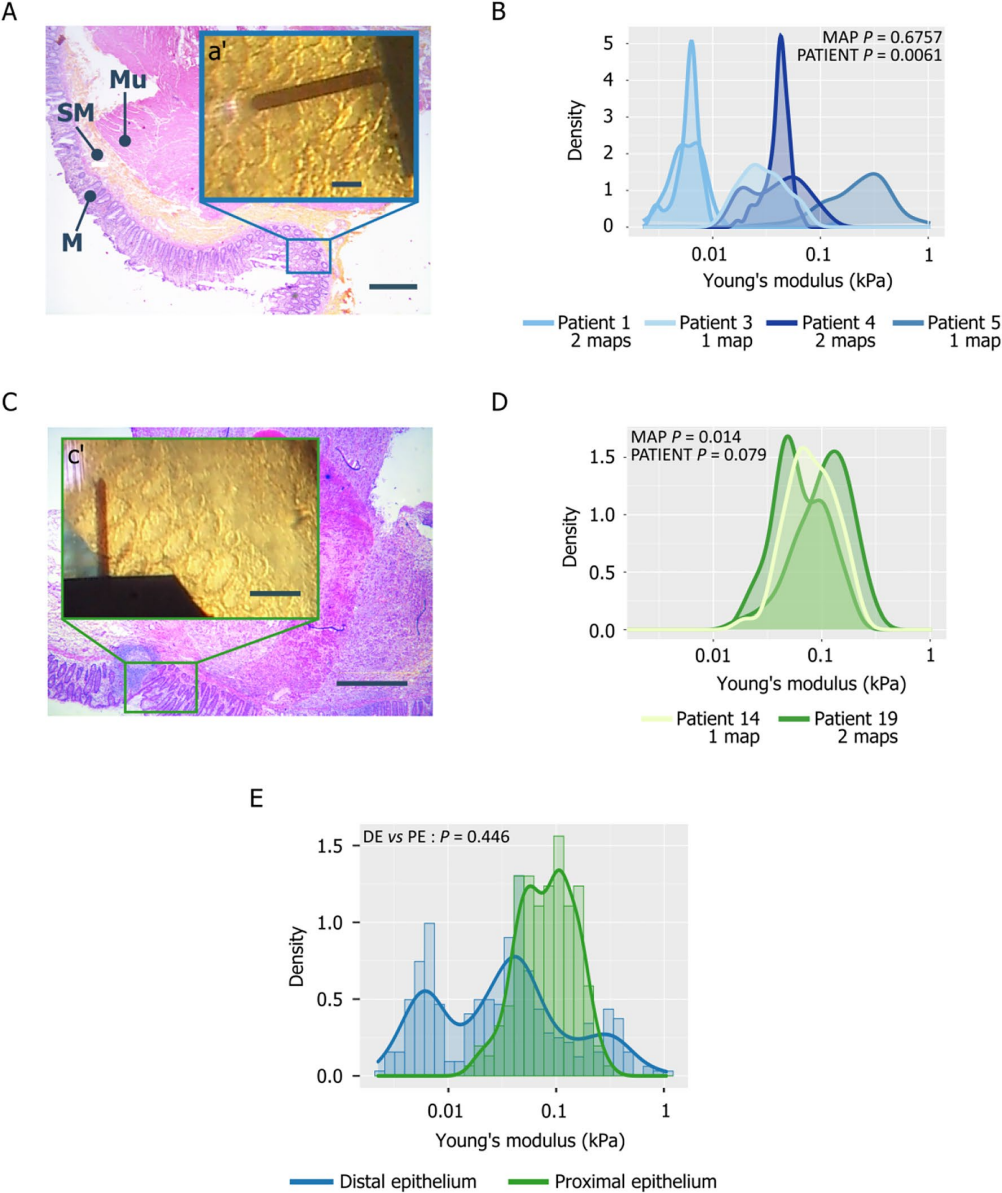
Rigidity of normal distal and proximal colon epithelium samples. (**A**) HES staining of normal colon: (M = mucosa, SM = submucosa, Mu = muscularis, bar = 500 µm); the inset corresponds to the same tissue area recorded with the top view camera of the AFM system (bar = 100 µm). (**B**) Histogram showing the Young’s modulus (kPa) of the mucosa layer for patients P1, P3, P4 and P5 (Log_10_ scale). (**C**) HES staining of anatomically normal tissue proximal to the tumor area (bar = 500 µm); the inset corresponds to the same area observed with the top view camera of the AFM system (bar = 100 µm). (**D**) Histogram showing the Young’s modulus (kPa) of normal proximal epithelium samples (Log_10_ scale) of patients P14 and P19. (**E**) Histogram showing the Young’s modulus of all distal and proximal healthy epithelium samples (Log_10_ scale). The *P*-values presented are extracted from Table 2 (*P*-values of MAP and PATIENT) and Table 3 (Tissue comparisons).

The colon wall is composed of four layers (Fig. 2A). The inner layer (mucosa) delineates the lumen and rests on the basement membrane. It consists of a single layer of rectangular cells forming an epithelium. The second layer (submucosa) contains blood and lymph vessels, nerves and loose connective tissue. The third layer (muscularis) is the muscular layer, and the fourth layer (serosa or adventitia) acts as a protective outer “skin” for the colon (not visible in Fig. 2A). The top view camera allowed centering the AFM cantilever precisely over the mucosa (Fig. 2a’) of non-transformed specimens resected far away from the tumor region (normal distal samples). Maps from the same patient were not significantly different ($p=0.6757$, Table 2) (i.e. homogeneous intra-patient stiffness of the distal normal epithelium). Conversely, maps from different patients displayed significantly different values ($p=0.0061,$ Table 2) (Fig. 2B). This indicated that the normal tissue stiffness value is specific for each patient.

Next, we studied the proximal “anatomically ”normal mucosa, which is sometimes observed at the tumor margins (Fig. 2C). This analysis gave good quality results only in samples from Patients 14 and 19. The maps of Patient 19 were significantly different ($p=0.014$) and this intra-individual variability did not allow differentiating Patient 19 from Patient 14 ($p=0.079$, Table 2, Fig. 2D). Moreover, by taking into account all intra- and inter-individual variations (Table S1), we could not differentiate between proximal and distal normal tissue maps ($p=0.446$, Table 3, Fig. 2E). Therefore, we pooled the normal distal and proximal epithelium data for comparison with the two other tissue types.

### Stiffness of colon tumors

**Figure 3:**
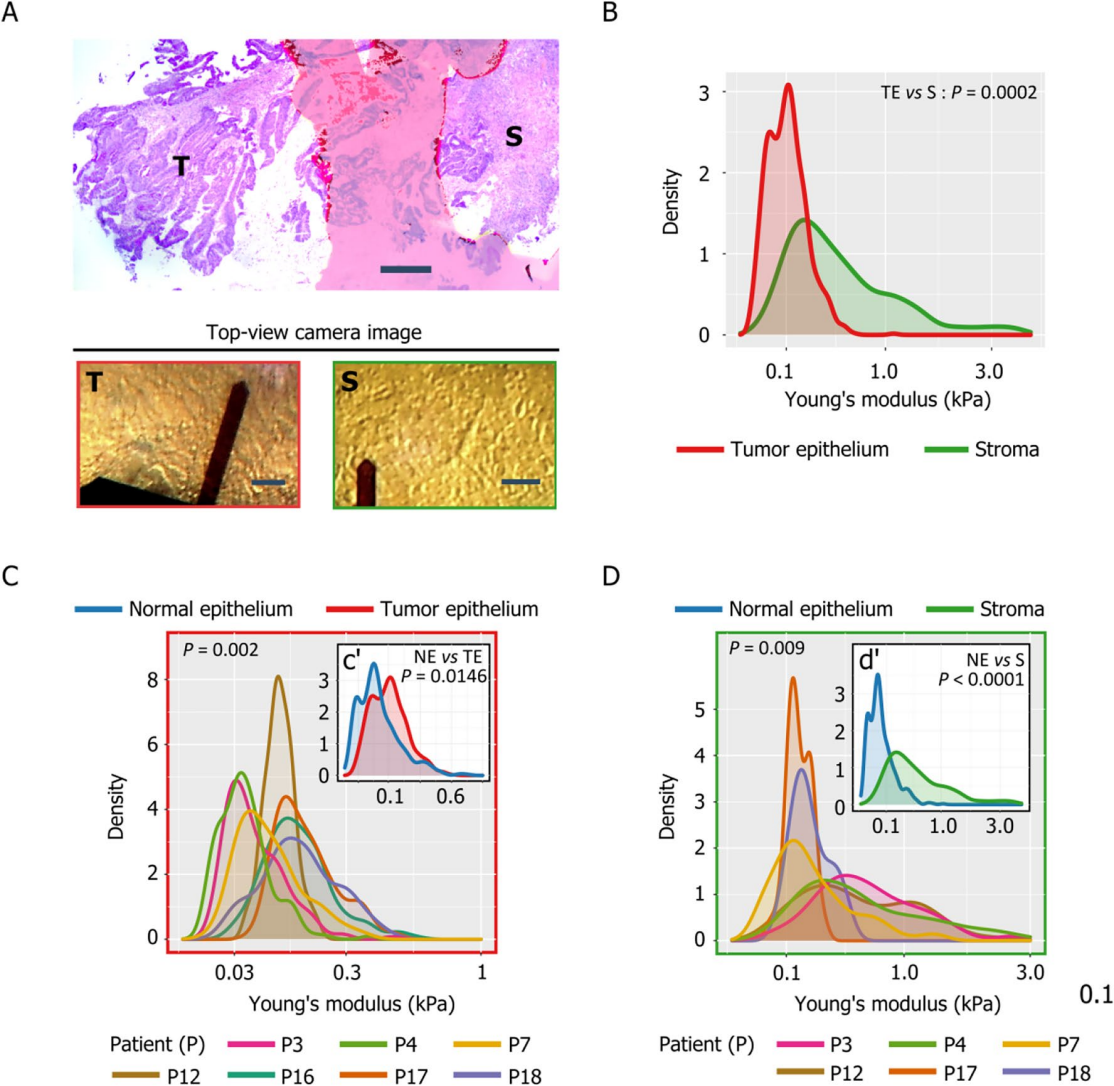
Stiffness of tumor epithelium- and stroma-rich regions of CRC samples: (**A**) HES staining of a CRC sample showing distinguishable tumor epithelial (T)- and stroma (S)-rich regions (bar = 500 µm) and the corresponding top view camera images (bar = 50 µm). (**B**) Histogram showing the Young’s modulus of epithelial-rich (red) and stroma-rich (green) regions of a CRC sample. (**C**) Histogram showing the Young’s modulus of the tumor epithelial-rich regions for all patients, and (c’) Histogram showing the Young’s modulus of tumor epithelial-rich regions (red) and normal epithelium (blue). (**D**) Histogram showing the Young’s modulus of the tumor stroma-rich regions of all patients and (d’) Histogram showing the Young’s modulus of tumor stroma-rich regions (green) and normal epithelium (blue). The *P*-values presented are extracted from Table 2 (*P*-values of MAP and PATIENT) and Table 3 (Tissue comparisons).

After HES staining, we could clearly spatially identify regions enriched in tumor epithelial cells (T) or in stromal cells (S) in seven and six CRC samples respectively (Fig. 3A). The top view camera allowed positioning the AFM cantilever tip specifically on the T or S (Fig. 3A) areas. After taking into account the intra- and inter-individual variability, the stiffness map profiles of these two areas were significantly different (Fig. 3B), with more scattered and higher stiffness values in S than in T regions ($p=0.0002$, Table 3, Supplementary Table 1). Moreover, values were significantly different among patients for both T (Fig. 3C, Supplementary Fig. 2A, $p=0.002$, Table 2) and S (Fig. 3D, Supplementary Fig. 2D, $p=0.009$, Table 2) regions.

Comparison of all maps obtained for each patient showed that the S region maps of the same patient were significantly different ($p=0.001$, Table 2) (Supplementary Fig. 2D) but not the T region maps ($p=0.0542$, Table 2) (Supplementary Fig. 2A). Comparison of the T and S maps and the normal tissue maps (Fig. 3c’, Supplementary Fig. 2B for T areas; Fig. 3d’, Supplementary Fig. 2E for S regions) indicated that stiffness values were higher in both T and S areas compared with normal tissues ($p=0.0146$ for T regions; $p<0.0001$ for S regions; Table 3, Supplementary Table 1). Moreover, the stiffness distribution profile of T areas, but not of S areas, was similar to that of normal areas, but with higher stiffness values (Fig. 3c′).

**Figure 4:**
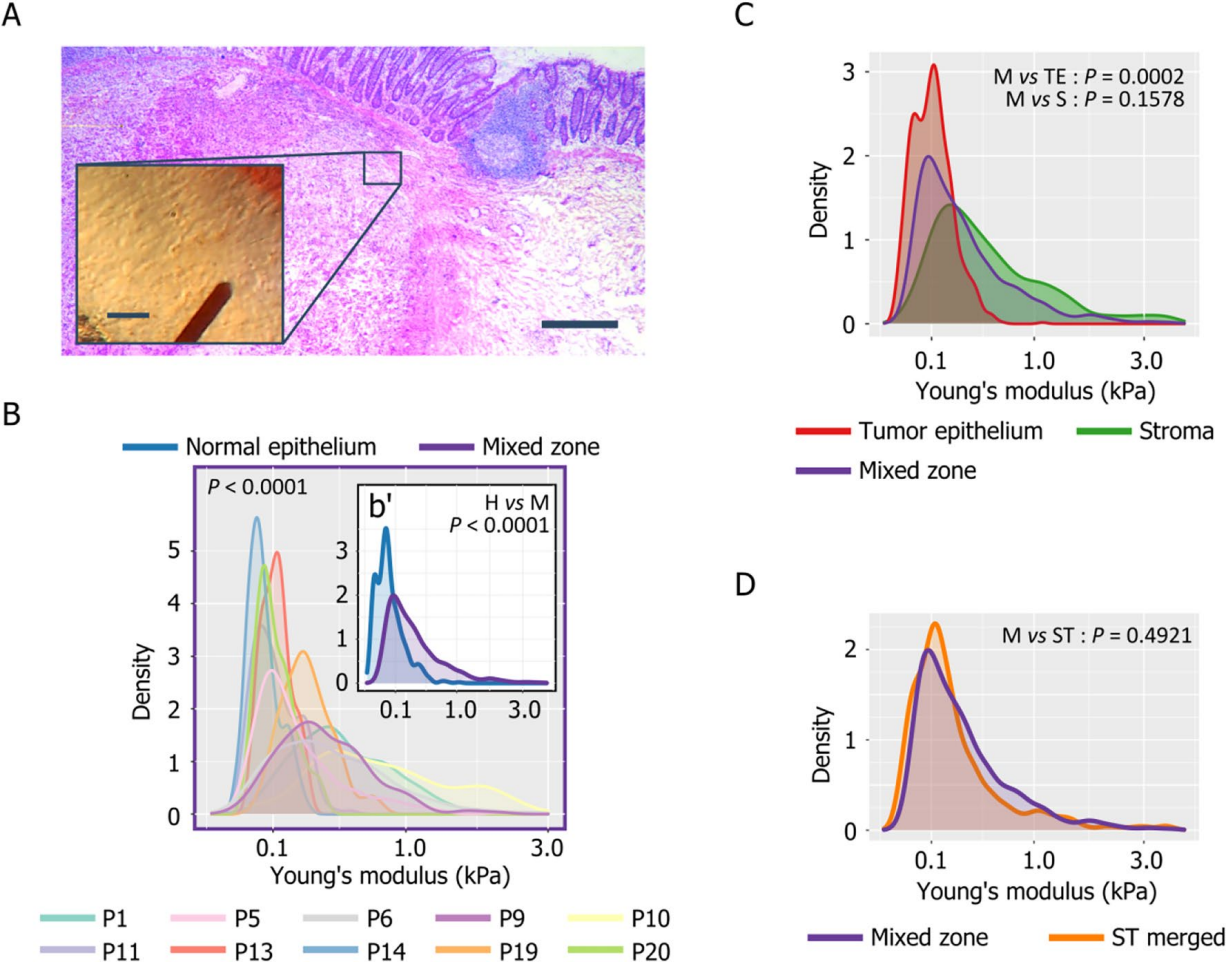
Stiffness of mixed CRC tissues (**A**) HES staining of a CRC sample showing a region in which epithelium- and stroma-rich areas cannot be clearly distinguished (i.e*.* mixed zone, bar = 500 µm) and in the inset the same region viewed with the top view camera of the AFM system (bar = 50 µm). (**B**) Histogram showing the Young’s modulus of mixed zones, b’) Histogram showing the Young’s modulus of mixed zones (purple) and normal epithelium (blue). (**C**) Histogram showing the Young’s modulus of tumor epithelium-rich (red), stroma-rich (green), and mixed (purple) regions. (**D**) Histogram showing the Young’s modulus of stroma- + epithelial-rich regions (ST Merged; orange) and mixed zones (purple). The *P*-values presented are extracted from Table 2 (*P*-values of MAP and PATIENT) and Table 3 (Tissue comparisons).

In the other ten CRC samples, we could not clearly separate the T and S regions on HES-stained sections (Fig. 4A).

Therefore, in these samples, we measured tissue stiffness in mixed zones that contain both tumor epithelial and stromal cells (Fig. 4A). Despite the significant intra-individual stiffness variability (Supplementary Fig. 3A) ($p<0.0001$, Table 2), we observed patient-specific profiles (Fig. 4B), as indicated by the significant inter-individual variability ($p<0.0001$, Table 2). Moreover, mixed zones were stiffer ($p<0.0001$, Table 3, Supplementary Table 1) than normal epithelia (Fig. 4b’) and also T regions ($p<0.0001$, Table 3, Table S1), but not S regions ($p=0.1578$, Table 3, Table S1) (Fig. 4C). However, when we pooled the S and T region data (S/T merged), we did not observe any significant difference between mixed zone and S/T merged stiffness profiles ($p=0.492$, Table 3, Table S1, Fig. 4D).

Lastly, analysis of the possible links between tumor tissue stiffness (S/T merged combined with mixed zone values) and clinical or molecular parameters (Table 4) showed that tumor location, stage, RAS mutational status, and perinervous infiltration were related to stiffness.

**Table 4:** GLMMPQL analysis of tumor tissue stiffness and clinical or molecular parameters.

Variables	n	$\beta$ parameter estimation	$t$*-*test $P$-value
**Clinics**			
Sex (ref = F)	36	− 0.098	0.6627
CRC Location (ref = Left)	29	− 0.564	0.0097
Age		− 0.0128	0.2425
**Pathological characteristics**			
Mucinous	26	− 0.356	0.1129
Lymphovascular invasion	37	0.2676	0.243
Perinervous Infiltration	22	0.625	0.005
Stage III + IV (ref = I + II)	41	0.90	< 0.0001
**Molecular characteristics**			
*KRAS* mutations	24	0.408	0.0753
*NRAS* mutations	3	− 0.794	0.3023
*RAS* mutations	27	0.498	0.0248
*BRAF* mutations	14	0.107	0.687
*RAS* + *BRAF* mutations	38	− 0.314	0.1656
Microsatellite status (ref = MSI)	28	0.291	0.1969

Tumor epithelium and stroma data were merged and combined with the mixed zone data (clinical or molecular data: fixed effects). The results of the fixed effects of the clinical features on 62 force maps are presented with their estimated *β* parameter. The associated *t*-test *p*-value and n represents the number of analyzed maps for each described variable or for the reference (ref) are also presented.

## Discussion

The mechanical response is increasingly considered as an important contributing factor in cancer and the associated tumor tissue stiffness is a major physical trait that could contribute to disease progression and treatment resistance in some cancers^38^. Therefore, it is important to better characterize stiffness in normal and transformed cells and tissues^39^. AFM micro- and nano-indentation methods are accurate tools for assessing these mechanical properties and are now becoming popular in cancer research^40,41^. However, the quantitative measurement of cell elasticity using the Young’s modulus in whole tissues is still challenging, and only few works have analyzed cancer samples^15,22,42^. First, tissues must be carefully collected, selected, annotated, cut, and preserved. Fresh tissues should be preferred, but this is very constraining. Alternatively, we and others^21^ showed that tissue preservation in OCT is a promising and applicable method. Second, the AFM setup must be adapted with a stage that has a large z-displacement (e.g. CellHesion stage from NanoBruker) to accommodate corrugated and sticky samples, such as tumor tissue specimens. Third, the stiffness of the AFM cantilever equipped with a 10 µm bead must be carefully equilibrated and calibrated in a liquid environment. Fourth, the highest possible number of force curves needs to be acquired to analyze tissue heterogeneity. Fifth, the AFM cantilever must be correctly positioned on the selected ROIs. We solved this issue by equipping the AFM with a top view optical setup that allowed us to measure the stiffness of sub-regions previously identified by histological examination, and then to compare different tissue structures. As the tissues analyzed came from CRC surgical specimens, we also integrated the patients’ clinical data in this proof-of-concept study.

The histology of colon tissue is well known; however, the stiffness of normal colon has never been assessed. To determine the mechanical properties of healthy colon, we measured the mucosa rigidity in normal (non-malignant) samples. We found that colon mucosa was very soft, less than 0.1 kPa on average, as reported for other normal tissues (e.g. breast gland and lung)^15^. The stiffness value of normal tissue tended to be different in each patient, highlighting the existence of inter-individual variability. Future studies might allow linking this variability to specific clinical features.

We then measured stiffness in tumor areas. These areas are composed of over-proliferating malignant epithelial cells (T) and of stromal cells (S) that could be clearly identified in some tumor sections after HES staining. We found inter-individual variability in the stiffness values of T regions, suggesting that the inter-variability observed in normal epithelial tissue remains also in the tumor. In S areas, stiffness values showed a strong intra-individual variability, and also inter-variability, for some patients, as previously observed in breast^43^ and pancreatic^41^ tumors. The stroma contribution to the higher stiffness observed in tumor tissues has been well described for breast^15^ and pancreatic cancer^44^, and may be implicated also in CRC^45^. Moreover, T areas were stiffer than normal distal and proximal areas, suggesting that tumor epithelial cells also become progressively stiffer during carcinogenesis. This may be due to changes in the expression of proteins that promote carcinogenesis, and could occur in response to stroma stiffening. Indeed, stiffness affects matrix biomarker expression and cancer cell shape in breast cancer^46^. In addition, as the tumor grows, changes in the mechanical properties of the tumor microenvironment could lead to the activation of molecular pathways, such as the β-catenin and YAP/TAZ signaling pathways^43,45^ that have roles in mechanotransduction^47^. It will be interesting to measure the rigidity of epithelial cells at the migration front in tumors and to correlate these rigidity maps with YAP expression in tumor cells.

By assessing correlations between the stiffness values and clinical parameters, we found that tumor location, stage, RAS mutational status, and perinervous infiltration were linked to rigidity and that left tumors were stiffer than right tumors. This is interesting because we observed higher colon tissue stiffness in the normal distal epithelium (i.e. left side) (data not shown), and higher collagen content in left tumors (Supplementary Fig. 4). Moreover, clinical data indicate that tumor location influences prognosis and treatment selection^2–4^. It would be interesting to obtain more data, with the aim of targeting tumor rigidity with specific drugs.

The correlation with the CRC stage also is interesting because tumor classification and patient stratification must be optimized to improve CRC management. As reported for breast cancer, our results strongly suggest that stiffness increases with cancer progression. Indeed, stiffness values were higher in late-stage tumors (III and IV, n = 6) than early stages tumors (I and II, n = 11) (p < 0.0001, Table 4). This could be due to an increase of the stroma to tumor epithelium ratio that is an independent factor for survival in patients with CRC, like lymph node and tumor stage^48^. The correlation between stiffness and stage must be now confirmed in a larger cohort.

We also found a possible relationship between stiffness and *RAS* mutations: *RAS* mutated tumors were stiffer than non-mutated tumors. *RAS* mutational status is very important for CRC management. Indeed, patients with metastatic CRC harboring *KRAS* or *NRAS* mutations are excluded from targeted therapies with anti-EGFR drugs, such as Cetuximab^®^^7^, and among patients with non-mutated CRC, only half will benefit of this therapy. However, the molecular mechanisms of resistance to Cetuximab^®^ are not fully understood^49^. The tumor stiffness may affect the drug access to the tumor center by directly preventing its diffusion inside the tumor or by modifying the tumor neo-vascularization, as described for liver CRC metastases^22^. Therefore, it would be interesting to test how the tissue stiffness influences the delivery of anti-EGFR agents and other molecules^50^. This could be performed first in vitro using multicellular spheroids derived from CRC cell lines or from patient-derived xenografts grown in matrices with different stiffness, as already described for glioma^51^. Moreover, these data highlight that gene mutations may change the tissue mechanical properties, leading to activation of specific pathways, especially in the case of KRAS that is genetically linked to YAP^52^.

Interestingly, a recent study supports the existence of a relationship between stiffness and genetic, notably KRAS, in mouse intestinal-derived cells^53^.

Perinervous infiltration also was linked to stiffness. This could be another interesting parameter because the interaction between tumor cells and neurons is associated with tumor aggressiveness and resistance to chemotherapy or radiotherapy^54^. Invasion of nervous structures by cancer cells is recorded by the pathologist and its detection is an independent predictor of poor outcome in CRC^55^. However, we did not observe nervous fibers in the samples used for AFM. Among the five tumors with perinervous infiltration recorded by the pathologists, four were excised from the left colon. To better understand the real link between stiffness, perinervous infiltration and tumor localization more experiments are needed.

## Conclusions

Altogether, our data demonstrated the feasibility of analyzing tissue rigidity from clinical samples by AFM. Our findings indicated that colon tissue is soft and becomes stiffer during carcinogenesis, particularly in stromal areas. Moreover, tumor stiffness seems to be positively associated with left colon localization, later stages, and RAS mutations. Additional studies in larger cohorts are needed to confirm these observations. Nevertheless, they suggest that targeting tumor stiffness with drugs, such as lysyl oxidase inhibitors, may improve treatment access in RAS-mutated CRC.

## Supplementary Information

Supplementary Information.

## Data Availability

The datasets used and/or analyzed during the current study are available from the corresponding author on reasonable request.
