# Supplementary material for: Mechanical signatures of human colon cancers
Source: Sci Rep. 2022 Jul 21;12:12475. doi: 10.1038/s41598-022-16669-3 (PMC9304395; doi:10.1038/s41598-022-16669-3)
Supplement: Supplementary file 1 — Supplementary Information. [file 41598_2022_16669_MOESM1_ESM.pdf]

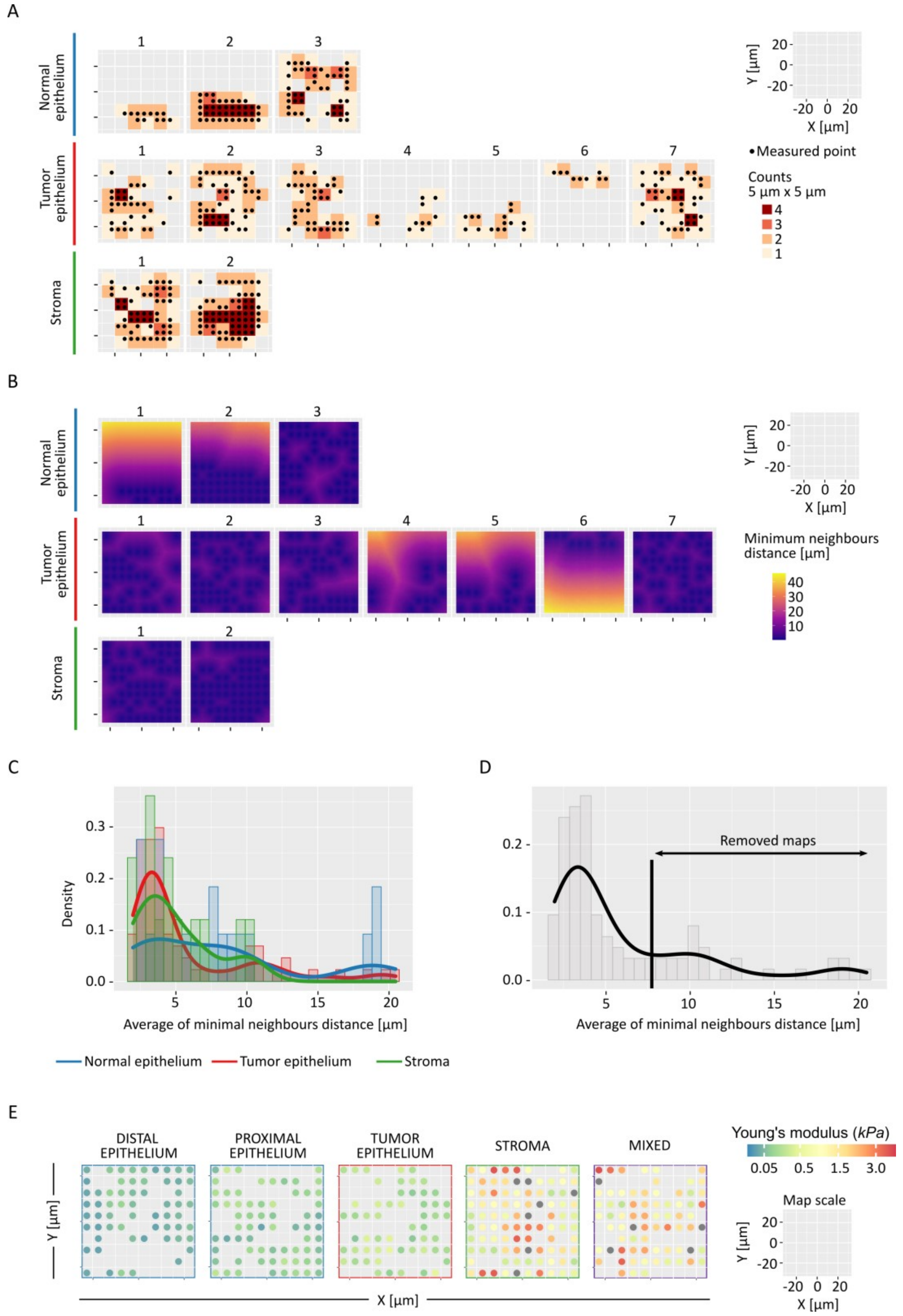

Figure S1: Map selection: Method to calculate the map quality score. For each map, **A)** the frequency of data per 10μm² squares and **B)** density were estimated. The nearest point was then calculated relative to the distance to the border in the 50 μm x 50 μm ROI. The mean value was used for each map. **C)** No difference in the quality score distribution was observed among the indicated tissue subtypes. **D)** A threshold of 8 units was used to remove all map with a value higher of this cut-off. **E)** Example of the density maps obtained.

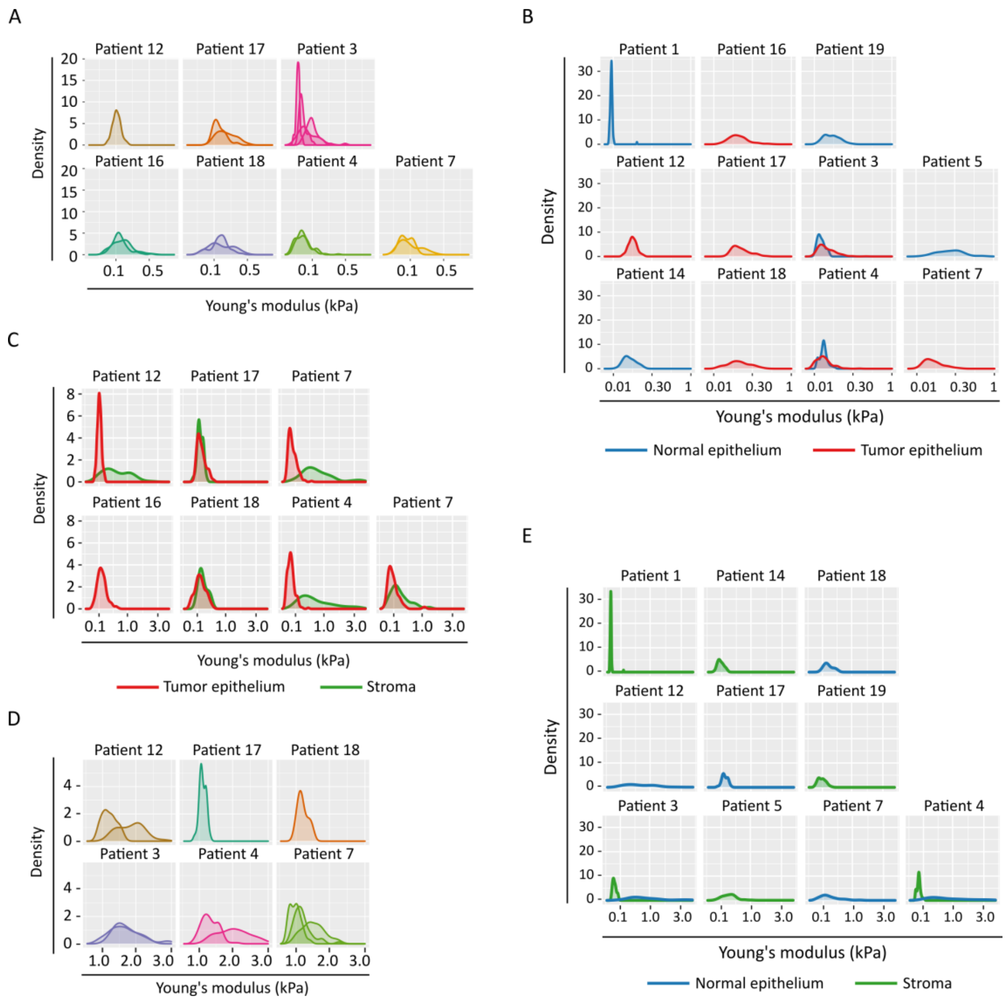

Figure S2: **Analysis of tumors with clearly distinguishable epithelial- and stroma-rich regions A)** Histograms showing the Young's modulus for the tumor epithelial-rich regions in the indicated patients. **B)** Histograms showing the Young's modulus of the tumor epithelial-rich region (red) and normal epithelia (blue) from the same patient, when available. **C)** Histograms showing the Young's modulus of the tumor epithelial-rich region (red) and stroma (green) of the same patient, when available. **D**) Histograms showing the Young's modulus of tumor stroma-rich regions. **E)** Histograms showing the Young's modulus of tumor stroma-rich region (green) and normal epithelia (blue) of the same patient, when available

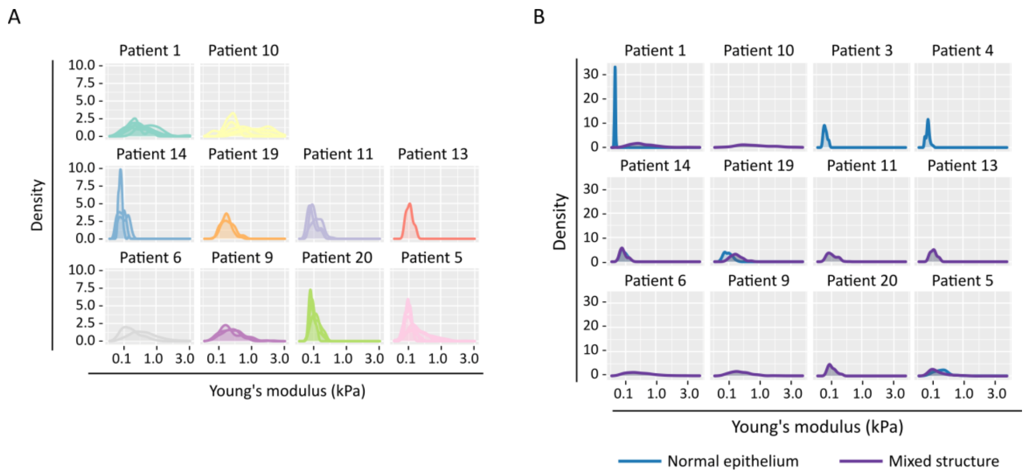

Figure S3: Data for all tumors with mixed epithelial and stroma regions. **A)** Histograms showing the Young's modulus of mixed structures in tumors. **B**) Histograms showing the Young's modulus of mixed structures in tumors (purple) and normal epithelia (blue) of the same patient, when available

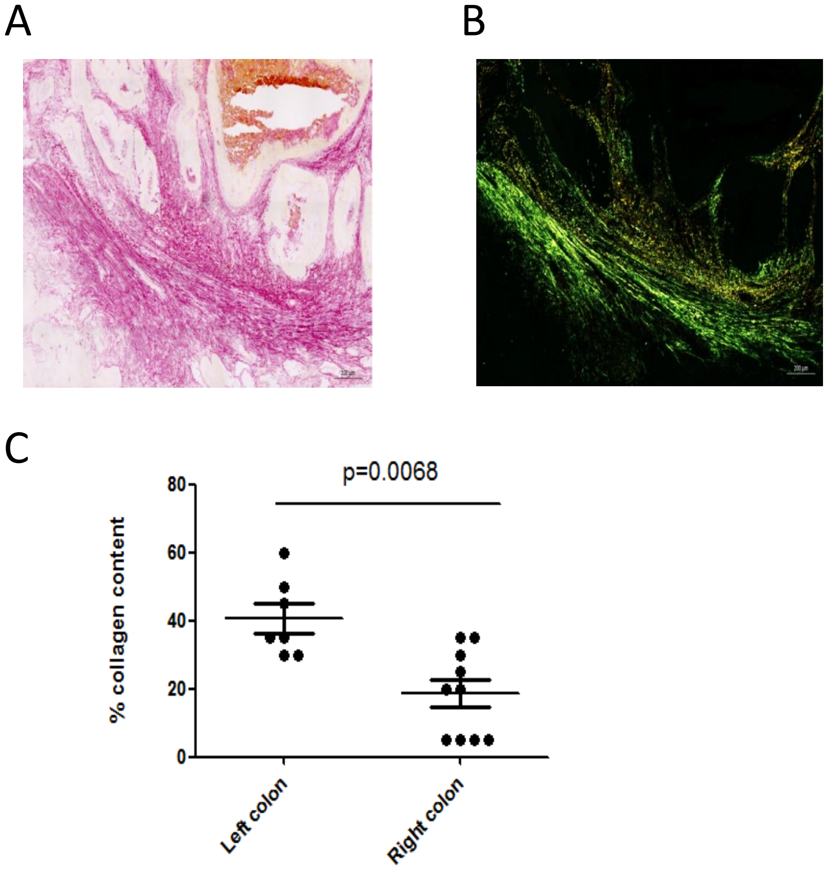

Figure S4: Collagen content analysis: After Picro-Sirius red staining of tumor specimens, collagen fibers were observed with a bright field illumination light microscope **A)** or a polarized light microscope **B); C)** Percentage of collagen fibers in function of the tumor location.

	Spatial model (GLMMPQL)					
	βcoef	SD	*t-value*	*P*	Chisq|Lratio	*P*
**Distal *Vs.* Proximal**					0.715	0.3977
Proximal	0.797	0.944	0.844	0.446		
Random						
Patient					5.96	0.0146
Map					5.96	0.0146
Map~subtype					9.47	0.0237
**Normal *Vs.* Tumor Epithelia**					5.986	0.0144
Tumor epithelium	0.192	0.078	2.44	0.0146		
Random						
Patient					7.91 (df 7,8)	0.0049
Map					7.91 (df 5,8)	0.048
Map~subtype					7.91 (df 5,8)	0.048
**Normal epithelium *Vs.* Stroma**					24.125	<0.0001
Stroma	2.18	0.445	4.91	<0.0001		
Random						
Patient					1.92	0.1654
Map					1.90E-04	0.989
Map~subtype					139.9	<0.0001
**Normal epithelium *Vs.* Mixed**					17.78	<0.0001
Mixed	1.75	0.415	4.214	<0.0001		
Random						
Patient					3.14	0.0763
Map					<0.0001	0.9996
Map~subtype					19.3	0.0002
**Tumor epithelium *Vs.* Stroma**					14.432	0.0001
Stroma	1.249	0.329	3.8	0.0002		
Random						
Patient					<0.0001	0.999
Map					<0.0001	0.999
Map~subtype					350.7	<0.0001
**Stroma *Vs.* Mixed**					2.066	0.1506
Mixed	-0.407	0.283	-1.437	0.1578		
Random						
Patient					<0.0001	0.999
Map					10.3	0.0013
Map~subtype					0.419	0.9364
**Tumor epithelium *Vs.* Mixed**					20.136	<0.0001
Mixed	0.969	0.216	4.486	<0.0001		
Random						
Patient					<0.0001	0.999
Map					6.72	0.0096
Map~subtype					34.503	<0.0001
**Tumor+Stroma *Vs.* Mixed**					0.4793	0.4887
Mixed	0.164	0.237	0.692	0.4921		
Random						
Patient					<0.0001	0.999
Map					6.57	0.0104
Map~subtype					5.55	0.136

Supplementary Table 1: **Results of the GLMMPQL analysis for each tissue**. The detailed models were summarized in Table 3: tissue comparisons with tissue type as fixed effect, β parameter and standard deviation (SD), and t test with the associated P value). Each comparison is detailed with the involved intra-individual variability (random Map effects) and inter-individual variability (random Patient effects) and the potential interaction between intra-individual variability and the tissue type used for comparison, as done in Table 2. They are presented with the resulted P values of the F test (F) for normal vs. tumor epithelia and the Likelihood ratio (Lratio) compared with the null model for the other comparisons.
